# Clinical relevance and governance of scout imaging data: a national survey of UK cross-sectional imaging radiographers

**DOI:** 10.1186/s13244-026-02306-4

**Published:** 2026-06-20

**Authors:** Theophilus N. Akudjedu, Julianna M. Hapi, Wisdom S. Mensah, Derrek A. Pinto, Benard Ohene-Botwe, Filson Kapilya

**Affiliations:** 1 Institute of Medical Imaging & Visualisation, School of Allied Health & Exercise Science, Faculty of Health, Environment & Medical Sciences, Bournemouth University, Bournemouth, UK; 2 University Hospitals Dorset, Bournemouth, UK; 3 Department of Midwifery & Radiography, City St George’s University of London, London, UK

**Keywords:** Scout imaging, Radiology management, Radiographers, Diagnostic imaging, Clinical governance

## Abstract

**Objectives:**

To examine the presence and content of standard policies and protocols for managing scout imaging data from cross-sectional imaging in the UK, including storage practices and required actions when clinical abnormalities are identified. The aim was to characterise current clinical processes and develop recommendations to address policy and knowledge gaps, thereby improving practice.

**Materials and methods:**

An online survey was distributed to cross-sectional radiographers in the United Kingdom between 23 May and 3 July 2022 using purposive sampling. Descriptive statistics were used to summarise quantitative data, and thematic analysis was applied to free-text responses.

**Results:**

Of 130 survey returns, 129 were valid. Most respondents worked in the NHS (83/129; 64.3%) and in England (104/129; 80.6%). The majority of cross-sectional radiographers (77/129; 59.7%) reported that all scout images are stored with the diagnostic image series. Overall, 61.2% (79/129) indicated that they had previously flagged abnormal findings on scout images. However, 45.7% (59/129) reported that their departments did not have clear policies and/or protocols governing the management of scout imaging data.

**Conclusions:**

Scout images may contribute to the detection of some abnormal findings. Nonetheless, the absence of clear local policies and protocols, and/or limited awareness of existing joint guidance, restricts their effective integration into diagnostic workflows and treatment planning. These findings indicate an urgent need for formalised departmental policies and targeted educational initiatives to support awareness creation for consistent, safe and effective management of scout images across UK radiology departments.

**Clinical relevance:**

Scout imaging data may contain clinically relevant information that could inform patient care. Standardisation of the governance and the awareness and creation of policies for the management of scout images among the clinical radiology workforce is urgently required.

**Key Points:**

- Scout images may hold significant diagnostic information that could contribute to patient care.
- Variation in the governance and protocols for management of scout imaging data reflects limited awareness of existing local and national guidance.
- Radiology workforce education is critical to creating awareness of policies and governance for scout imaging data management in practice.

**Graphical Abstract:**

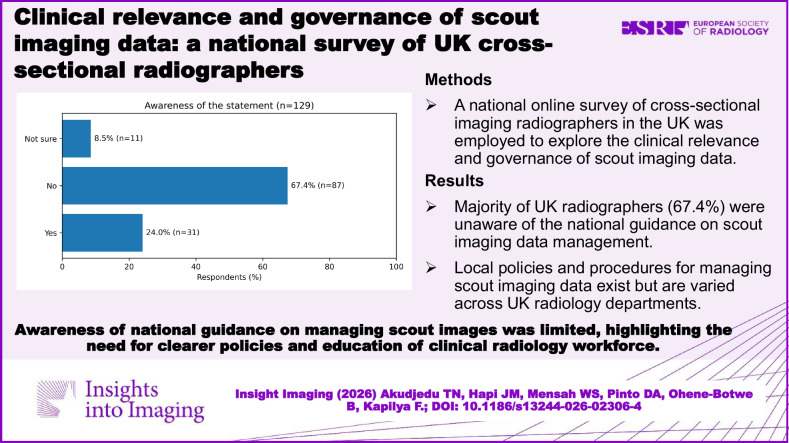

## Introduction

Advancements in cross-sectional imaging techniques, including computed tomography (CT) and magnetic resonance imaging (MRI), have significantly improved patient care by aiding diagnosis across a wide range of clinical conditions [1]. Although CT and MRI differ in their technical and operational principles, both employ a similar image acquisition process that begins with pre-planning localiser images, referred to as scout, pilot, surview, topogram, scanogram, or preview images, depending on the vendor or jurisdiction [2–5]. These images are essential for localising the anatomical region of interest and planning subsequent scans [6, 7]. Additionally, they provide technical insight for optimising acquisition parameters, such as matrix size in MRI or tube current modulation and z-dimension coverage in CT image acquisition [8]. Scout images also play a role in safety screening in some clinical scenarios. For example, a CT scout can be used to assess unconscious trauma patients before MRI, reducing the need for multi-view plain radiographs and helping to limit cumulative radiation exposure [9]. In some interventional procedures, scout images play a vital role in contrast injection or guidewire manipulation procedures [10].

Beyond localisation and screening, there is growing evidence [11–13] that scout images may hold clinically relevant information that supports some diagnostic process. Several studies have shown that abnormalities visible on scout images may not always be captured within the diagnostic series, particularly when they lie outside the primary area of interest [11–13]. For example, Thaker and colleagues reported that abdominal CT scout images revealed pathology at the lung bases and retained surgical foreign bodies that were not evident on diagnostic axial images [14]. Similarly, MRI scout images have been reported to display unexpected abnormalities, such as epidural masses, which influenced subsequent imaging decisions during surgical preparations [15]. Notwithstanding, scout images are often overlooked in routine practice. This has been associated with delayed diagnosis and adverse outcomes [15, 16], including a reported case of a missed fracture, on a paediatric head CT where the abnormality was visible on the scout image but not reviewed [17].

Despite this evidence, the management and review of scout images vary considerably across healthcare systems. In the United States, only a few American College of Radiology (ACR) guidelines explicitly require that scout images be stored or reviewed within the picture archiving and communication systems (PACS) [18]. There are no clear regulations mandating whether these images should be routinely stored or examined by radiologists [2]. In an anecdotal statement, an expert witness, Leonard Berlin [17], mentioned that up to three-quarters of radiologists either overlook or do not routinely include scout images in their CT interpretations. Compounding the issue is that scout images are often transferred into PACS as a separate series, which may reinforce this pattern, leading to what has been called ‘scout neglect bias’ [19, 20].

In the United Kingdom (UK), the role of scout images has been debated, partly due to concerns about their perceived lower diagnostic quality [21, 22]. In response, the Clinical Imaging Board (CIB) of the UK, including the Royal College of Radiologists (RCR), Society of Radiographers (SoR), Institute of Physics and Engineering in Medicine (IPEM), and College of Radiographers (CoR), issued a joint statement advocating for the inclusion of scout images in PACS and calling for clear, standardised protocols to ensure their consistent management [23]. They argue that these images can provide essential supplementary data for radiologists in the diagnostic pathway.

This study aims to evaluate how local policies and protocols for the management of scout images are currently implemented in UK radiology departments from the perspective of cross-sectional imaging radiographers, particularly following the publication of the joint professional statement [23]. It also examines radiographers’ awareness of the policies and protocols, clinical significance of scout images and their integration into the diagnostic pathway. By identifying gaps in practice and policy, the study will support the development of local policies and protocols, enhance radiographers’ awareness of national guidance, and improve diagnostic quality, thereby promoting safer and more effective patient care.

## Methods

### Survey design and distribution

An online cross-sectional survey targeting UK radiographers currently practising, managing, or leading cross-sectional imaging activities was conducted from May 23 to July 3, 2022. The study employed a pragmatic purposive sampling approach based on a relatively broad inclusion criterion to include participants who are qualified Radiographers with direct professional experience in CT and/or MRI practice and service management roles relevant to scout image acquisition, review and data management. Thus, all cross-sectional imaging radiographers with any amount of professional experience are eligible. The survey questionnaire, which was developed following a review of the literature, aimed to gather information on four key areas: (1) participants’ demographics, (2) management of scout images, (3) awareness of local and national policies from the CIB about scout image management, and (4) the clinical significance of scout images. The questionnaire included both closed—and open-ended questions. To enhance understanding and minimise ambiguity, the survey questions were drafted using a simple syntax. Content validity was established through expert review by two academic radiographers, and the instrument was piloted with three practising radiographers, whose feedback informed minor revisions before distribution. The survey was hosted on Google Forms, and the survey link was shared via email with Radiology leads from National Health Service (NHS) Trusts and radiology managers from the private sector across the UK, to be distributed to their staff. Additionally, it was promoted via social media platforms to maximise response rates. The survey response timeframe was 6 weeks, during which weekly reminders were sent on social media.

Data collection encompassed radiographers from various settings across the UK, including the NHS, academic institutions, private practices, and mobile van operations. This diverse range of sites was selected to ensure robust data, as each department operates under different protocols and procedures. Participants not engaged in cross-sectional imaging were excluded from the study. This was ensured by providing clear information alongside the survey link about the participants needed for this study and by including filter questions to screen out ineligible respondents. Before participation, each respondent received an explanation of the study, including its purpose, procedures, potential risks, and benefits. Informed consent was obtained via an electronic consent form, in which participants confirmed their understanding and agreement to participate by ticking a checkbox or providing a digital signature. The form also assured participants of their anonymity, confidentiality of their responses, and their right to withdraw from the study at any time without penalty. To maintain anonymity, codes were generated to represent each participant. The Research and Ethics Committee of Bournemouth University approved the study (Ethics ID: 43852).

### Data analysis

The data collected via Google Forms were exported to the Statistical Package for the Social Sciences (SPSS) version 28.0 for Windows for analysis. Descriptive statistics were used to summarise the data in terms of absolute numbers and frequencies. Chi-square (χ2) tests were used to examine associations between categorical variables, including work setting, geographical location, and awareness or management of scout images. A two-tailed α level of 0.05 was used to determine statistical significance. Cases with missing responses were excluded from the relevant analyses.

Qualitative data from open-ended survey questions were analysed using thematic analysis [24], following the six-phase approach described by Braun and Clarke [25]. This included: (1) familiarisation with the data, (2) generating initial codes, (3) searching for themes, (4) reviewing themes, (5) defining and naming themes, and (6) producing the thematic report. Coding was conducted manually by the lead researcher into an Excel form, and a second researcher independently reviewed it. Discrepancies in coding were discussed with the entire team until agreement was reached.

## Results

### Response rate and demographics

During the five-week data collection period, 130 responses were obtained; however, only 129 were considered valid, as one respondent did not complete the survey. Detailed demographic data are presented in Table 1. The respondents comprise CT radiographers (31/129; 24.0%), MRI radiographers (30/129; 23.3%), CT/MRI radiographers (35/129; 27.1%), Rotational radiographers (X-ray/CT) (13/129;10.1%), radiology/radiography manager/Lead (19/129; 14.7%) and others (1/129; 0.8%).

**Table 1:** Demographic distribution of respondents

Variable	Group	Frequency	Percentage
Age group	18–29	36	27.9%
	30–39	42	32.6%
	40–49	31	24.0%
	50–59	19	14.7%
	60+	1	0.8%
Professional status	CT	31	24.0%
	MRI	30	23.3%
	CT/MRI	35	27.1%
	Rotational	13	10.1%
	Radiology lead	19	14.7%
	Other(s)	1	0.8%
Geographical location	England	104	80.6%
	Wales	13	10.1%
	Scotland	7	5.4%
	Northern Ireland	5	3.9%
Years of experience	0–5	45	34.9%
	6–10	29	22.5%
	11–15	23	17.8%
	16+	32	24.8%
Work setting	NHS	83	64.3%
	Academic setting	16	12.4%
	Private	21	16.3%
	Mobile van	9	7.0%

The demographics of the respondents across settings and the UK nations

The majority of respondents worked in NHS settings (83/129; 64.3%) and private settings (21/129; 16.3%). Geographically, England had the highest number of respondents (104/129; 80.6%). In terms of age, 32.6% (42/129) were aged 30-39 years, and 34.9% (45/129) had 0–5 years of work experience in cross-sectional imaging. The study found significant relationships between work setting and geographical location (*p* = 0.01); however, no significant associations were found for age group (*p* = 0.70), professional status (*p* = 0.70), or work experience (*p* = 0.20).

### Management of scout imaging data across UK Radiology Departments

Across NHS Trusts (64/129; 49.6%) and university or academy settings (13/129; 10.1%), most cross-sectional radiographers reported that their departments store all diagnostic series, including scout images. A smaller proportion (9/129; 7.0%) indicated that their departments do not store scout images at all, while others noted that storage practices vary by examination.

A significant majority (82/129; 63.6%) stated that these images are retained for more than six months, particularly in England (68/129; 52.7%), followed by Wales and Scotland (both 5/129; 3.9%). However, 26.6% (34/129) of respondents were unsure of their department’s storage duration policy.

Most departments (123/129; 95.3%) transfer scout images to PACS, with responses primarily from England (98/129; 76.0%), followed by Wales (13/129; 10.1%), Scotland (7/129; 5.4%), and Northern Ireland (5/129; 3.9%). A small number of participants reported using alternative storage solutions, including other archiving systems (3/129; 2.3%), scanner computer drives (2/129; 1.6%), or external storage (1/129; 0.8%).

Regarding image transfer, 69.8% (90/129) of respondents indicated that localiser images are transferred automatically alongside the diagnostic series. A further 15.5% (20/129) reported manually transferring scout images, while 7.8% (10/129) were unsure of the process.

### Awareness of local and national policy from the CIB

Of the 129 respondents, 46.5% (60/129) confirmed that their departments have procedures for managing aborted examinations after acquiring scout images, while 35.7% (46/129) stated that they do not, and 17.8% (23/129) were unsure. Awareness of local policies was highest among radiographers working in NHS Trusts (40/129; 31%), followed by those in private settings (8/129; 6.2%), university or academy settings (7/129; 5.4%), and mobile van services (5/129; 3.9%). Notably, 15.5% (20/129) of respondents from England were unsure whether a departmental policy was in place.

**Fig. 1:**
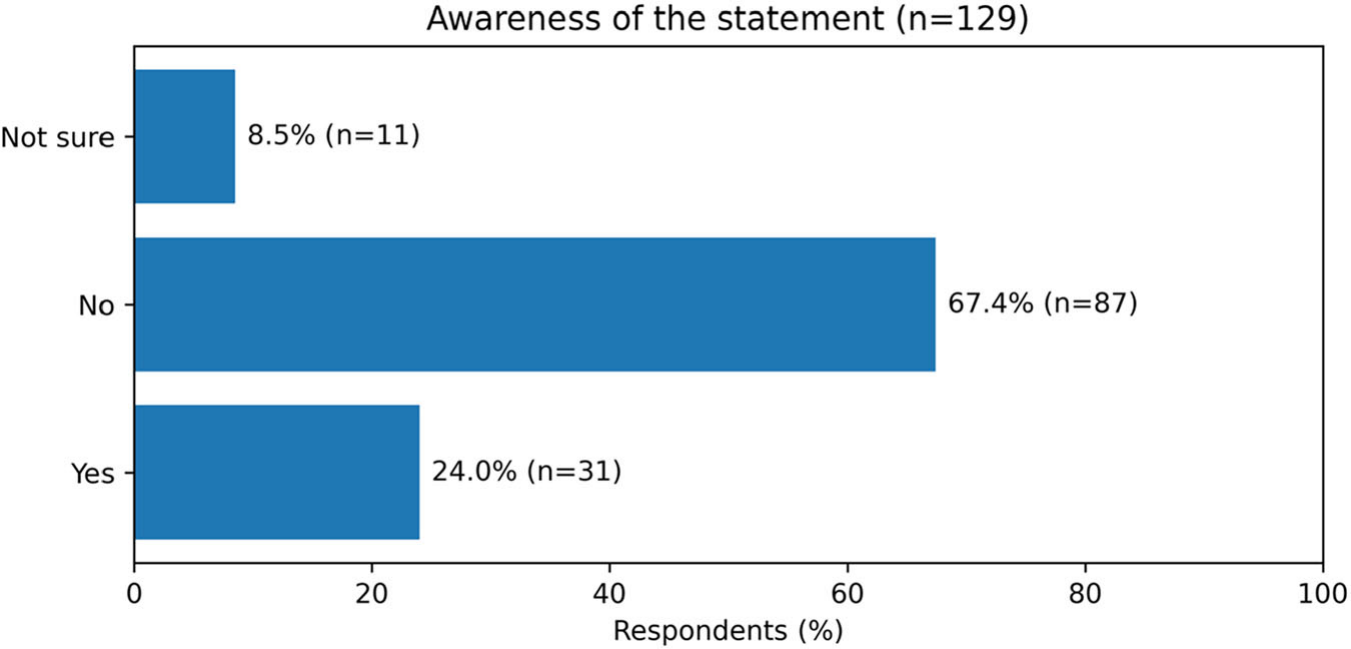
Awareness of the joint position statement from the CIB on scout imaging data management

**Fig. 2:**
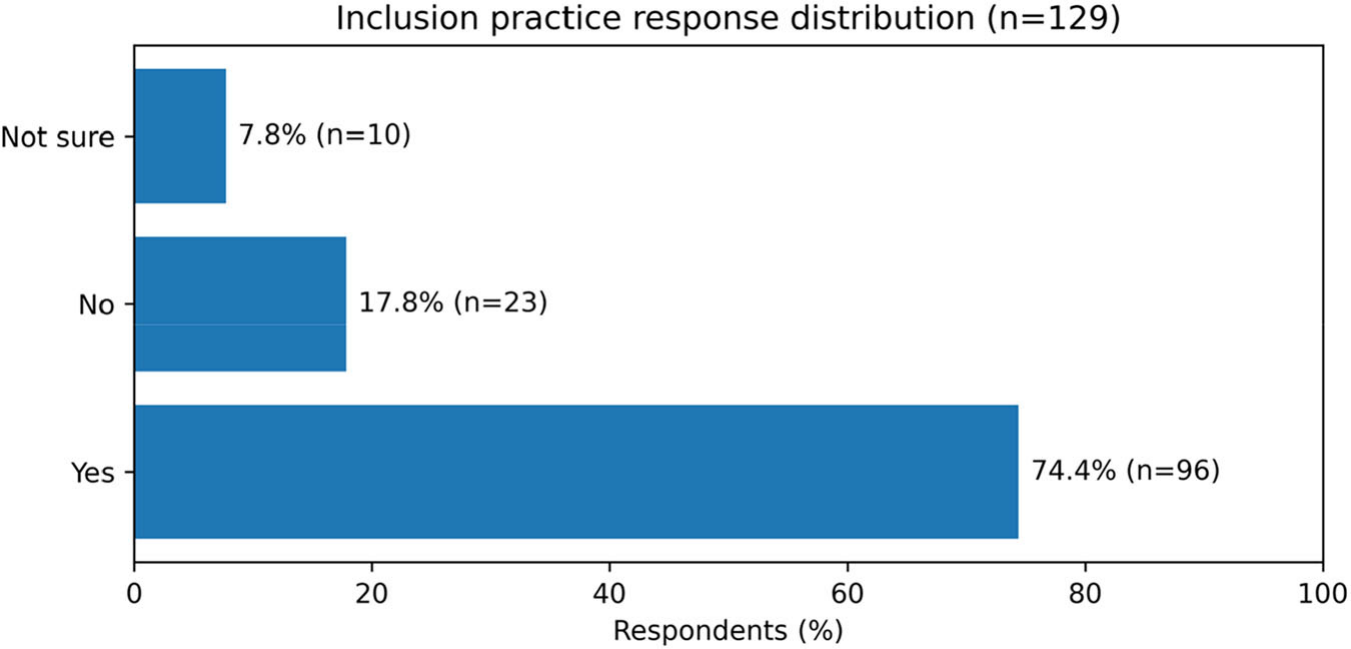
Inclusion of scout images in diagnostic series before transferring to PACS/other systems, before the release of the 2021 joint statement

Among those who reported having departmental policies, 36.4% (47/129) indicated that the existing policy does not clearly explain the rationale for including or excluding scout images. A further 39.5% (51/129) stated that no formal protocols exist (see Table 2). As shown in Fig. 1, only 24.0% (31/129) of respondents were aware of the CIB’s joint position statement on the management of scout images. In contrast, 67.4% (87/129) reported no awareness of the document, and 8.5% (11/129) were unsure. Figure 2 shows that 74.4% (96/129) confirmed that scout images were included in their diagnostic series before being transferred to PACS or other systems before 2021.

**Table 2:** Distribution of participant responses relating to the management of scout images

Question	Responses, *n* (%)				
Do you store acquired diagnostic series along with scout/localiser images at your department?	All together with diagnostic series	Diagnostic series only	Depending on the type of examination	Not sure	Other
Response across settings					
NHS	64 (49.6%)	6 (4.7%)	8 (6.2%)	3 (2.3%)	
Academic	13 (10.1%)	1 (0.8%)	2 (1.6%)	0 (0.0%)	
Private	12 (9.3%)	1 (0.8%)	4 (3.1%)	3 (2.3%)	
Mobile van	7 (5.4%)	1 (0.8%)	1 (0.8%)	0 (0.0%)	
Response across UK nations					
England	76 (58.8%)	8 (6.2%)	12 (9.3%)	5 (3.9%)	
Wales	9 (7%)	1 (0.8%)	2 (1.67%)	1 (0.8%)	
Scotland	7 (5.4%)	0 (0%)	0 (0.0%)	0 (0.0%)	
Northern Ireland	4 (3.1%)	0 (0%)	1 (0.8%)	0 (0.0%)	
How do you transfer your localiser/scout images for storage/reporting/post-processing at your department? Select all that apply.	Manually	Automatically	Not sure	Other	
Response across settings					
NHS	9 (7%)	70 (54.3%)	4 (3.1%)	0 (0.0%)	
Academic	4 (3.1%)	11 (8.5%)	1 (0.8%)	0 (0.0%)	
Private	5 (3.9%)	12 (9.3%)	4 (3.1%)	0 (0.0%)	
Mobile van	2 (1.6%)	5 (3.9%)	1 (0.8%)	1 (0.8%)	
Response across the UK nations					
England	18 (14%)	76 (58.9%)	9 (7%)	1 (0.8%)	
Wales	1 (0.8%)	11 (8.5%)	1 (0.8%)	0 (0.0%)	
Scotland	1 (0.8%)	6 (4.7%)	0 (0.0%)	0 (0.0%)	
Northern Ireland	0 (0.0%)	5 (3.9%)	0 (0.0%)	0 (0.0%)	
Total					
How long do you store your scout/localiser images on PACS or other archiving systems?	0–2 Months	3–5 months	6 months+	Not sure	
Response across settings					
NHS	4 (3.1%)	4 (3.1%)	51 (39.5%)	24 (18.6%)	
Academic	0 (0%)	1 (0.8%)	11 (3.9%)	4 (3.1%)	
Private	0 (0%)	1 (0.8%)	15 (11.0%)	5 (3.9%)	
Van	1 (0.8%)	2 (3.6%)	0 (0%)	1 (0.8%)	
Response across UK nations					
England	4 (3.1%)	6 (4.7%)	68 (52.7%)	26 (20.2%)	
Wales	0 (0.0%)	1 (0.8%)	5 (3.9%)	7 (5.4%)	
Scotland	0 (0.0%)	1 (0.8%)	5 (3.9%)	1 (0.8%)	
Northern Ireland	1	2(0	4 (3.11%)	0 (0.0%)	
Where do you transfer your cross-sectional imaging data for storage/reporting/post-processing at your department?	PACS	Other	Computer drive	External (third-party entity)	Not sure
Response across settings					
NHS	81 (62.8%)	1 (0.8%)	1 (0.8%)	0 (0%)	
Academic	14 (10.9%)	1 (0.8%)	0 (0%)	1 (0.8%)	
Private	21 (16.3%)	0 (0.0%)	0 (0%)	0 (0.0%)	
Mobile van setting	7 (5.4%)	1 (0.8%)	0 (0%)	0 (0.0%)	
Response across UK nations					
England	98 (76%)	3 (2.3%)	2 (1.6%)	1 (0.8%)	
Wales	13 (10.1%)	0 (0.0%)	0 (0.0%)	0 (0.0%)	
Scottland	7 (5.4%)	0 (0.0%)	0 (0.0%)	0 (0.0%)	
Northern Ireland	5 (3.9%)	0 (0.0%)	0 (0.0%)	0 (0.0%)	

Participants’ responses across settings and UK nations regarding the management of scout images in their departments

Of the respondents, 61.5% (79/129) reported previously flagging abnormal findings, whereas 27.7% (36/129) had not and 10.8% (14/129) were unsure (Table 3).

**Table 3:** Awareness of specific local and national policies from the CIB

	Yes	No	Not sure		
Existence of policies for the management of imaging data acquired after aborting examination.	60 (46.58%)	46(35.7%)	23(17.8%)		
Does your department currently have a local policy and protocol in place for the management of scout/localiser images?	Yes	No	Not sure		
Response across settings					
NHS	40 (31%)	24 (18.6%)	19 (14.7%)		
Academic	7 (5.4%)	7 (5.4%)	1 (0.8%)		
Private	8 (6.2%)	12 (9.3%)	2 (1.6%)		
Mobile van	5 (3.9%)	3 (2.3%)	1 (0.8%)		
Response across UK nations					
England	52(40.3%)	32(24.8%)	20(15.5%)		
Wales	5(3.9%)	5(3.9%)	3(2.3%)		
Scotland	1(0.8%)	6(4.7%)	0(0%)		
Northern Ireland	2(1.6%)	3(2.3%)	0(0%)		
If a protocol or policy exists, does it state why scout/localiser images should be included or excluded in a diagnostic set series?	Yes + rational for inclusion	Yes, but no rationale for inclusion	No	Not sure	Other
Response across settings					
NHS	7 (5.4%)	9 (7%)	31 (24%)	35 (27.1%)	
Academic setting	3 (2.3%)	2 (1.6%)	8 (6.2%)	2 (1.6%)	
Private	3 (2.3%)	2 (1.6%)	11 (7.8%)	6 (4.7%)	
Mobile van setting	3 (2.3%)	1 (0.8%)	2 (16%)	3 (2.3%)	
Response across UK nations					
England	13 (10.1%)	11 (8.5%)	39 (30.2%)	39 (30.2%)	
Wales	2 (1.6%)	0 (0%)	4 (3.1%)	7 (5.4%)	
Scotland	1 (0.8%)	1 (o.8%)	5 (3.9%)	0 (0%)	
Northern Ireland	0 (0.0%)	2 (3.6%)	3 (2.3%)	0 (0%)	
	Yes	No	Not sure		
Are you aware of the joint position statement by the RCR, SoR, IPEM, and the CoR in relation to the management of scout/localiser images?	31 (24%)	87 (67.4%)	11 (8.5%)		
Prior to 2021 (when this position statement was published), were you including scout/localiser images in the diagnostic series before transferring to PACS/other systems?	96 (74.4%)	23 (17.8%)	10 (7.8%)		

Participants’ responses across settings and UK nations regarding awareness of local and national policies on scout imaging

### Themes developed from the free-text commentary

The thematic analysis of the free-text responses yielded two main themes: policies for the management of scout images, and diagnostic value of Scout images (Table 4).

**Table 4:** Themes developed from the open-text commentary

Representative quotations	Sub-theme	Theme
*“I have read articles in SOR synergy etc regarding this, and I believe that scout, localiser images are just as important as the diagnostic ones”* (Case ID 40- MRI Radiographer) *“This study has brought to my attention the 2021 statement from the Clinical imaging Board”* (Case ID 20 -Radiology Manager) *“I have never questioned the reasoning behind including or not including the localiser.”* (Case ID 10, MRI Radiographer)	Awareness of professional guidance	*1*. Policies for the management of scout images
*“I will now draft a Standard Operating Procedure to manage abandoned exams and scout images.”* (Case ID 60, Radiology Manager) *“I now intend creating a policy to comply and back up our current procedures.”* (Case ID 20, Radiology Manager) *“There is a procedure, but it is not written down.”* (Case ID 2, CT Radiographer)	Documented policy	
*“I believe that scout images are an essential part of the diagnostic process and should be saved as a permanent PACS record and included in the reporting checklist of any reporter.”* (Case ID 11- MRI Radiographer) *“I have always believed that all unique sequences should be archived so they can be reviewed by a radiologist. While some sequences appear of little value (movement, low SNR etc.) only when combined with others can they create additional diagnostic information*. (Case ID 19- Radiology Manger) *“I don’t think localisers are considered much by many people but can be very useful.”* (Case ID 39, CT Radiographer) *“Very informative study. I will need to read more research on this topic.”* (Case ID 30, CT/MRI Radiographer)	Attitude of Practitioners	
*“Fracture of wrist on a TRAUMA CTCHAP.”* (Case ID 16, Rotational Radiographer) *“Perforation.”* (Case ID 33, CT/MRI Radiographer*)* “From seeing a suspected pathology on the scout image, I have run additional MRI sequences to appropriately investigate the pathology, for example T2* gradient echo (GRE) or susceptibility Weighted Imaging (SWI) in the suspicion of cavernous haemangeoma or haemorrhage.” - (Case ID 8- MRI Radiographer)	Suspected abnormalities	2. Diagnostic and clinical value of Scout images
*“Identifying incidental findings such as a time a significant mass in a kidney was identified on a coronal localiser which otherwise would not have been seen on the diagnostic images focussed on the region of interest referred for”*. (Case ID 3- Radiology Manager) *“MRI sagittal localiser for cervical spine showed tumour in cerebellum.”* (Case ID 18, CT/MRI Radiographer) *“Patient for cervical spine; spotted an abnormality in the brain on the localiser images and added some brain sequences. They confirmed the patient had an actual brain lesion.”* (Case ID1- MRI radiographer) *“Large effusion spotted on chest when scout performed for abdominal CT.”* (Case ID 2, CT Radiographer) *“1.Primary lung cancer spotted on spine MRI; 2. Renal cysts on spinal MRI; 3. Tibial tumour on knee MRI; 4. Unknown Pelvic kidney on spinal MRI; 5. Fibroids on spinal MRI”* (Case ID 4-MRI Radiographer) *“Abdominal aortic aneurysm and renal mass on lumbar spine MRI*.” (Case ID 28, MRI Radiographer)	Incidental findings on scout images	
*“Spotted numerous fractures on scout images that have led me to change my scan sequences and start the process of further management early on.”* (Case ID 6, MRI Radiographer) *“Extensive renal tumour seen on coronal scout… indicated additional sequences and arranged for on-call oncologist to view.”* (Case ID 33, MRI Radiographer)*“Several instances of abdominal/spinal abnormalities… leading to further imaging, including several abdominal aortic aneurysms.”* (Case ID 45, MRI Radiographer)	Influence of incidental findings	

Some direct participant quotations that informed themes. Case ID refers to the anonymised identifier assigned to each respondent

#### Theme 1: awareness of local and national policies and management of scout images

Some participants were familiar with professional guidance, including the CIB’s joint position statement. However, a significant number of radiographers were unaware of national recommendations or uncertain whether local protocols existed in their departments. Several comments suggested that informal practices were in place but lacked formal documentation, and several respondents expressed interest in developing or learning more about appropriate governance processes.

#### Theme 2: diagnostic and clinical value of scout images

The responses highlighted the diagnostic contribution of scout images beyond their traditional role in localisation. Participants described a range of abnormalities visible on CT and MRI scout images, including brain, renal, hepatic and gynaecological abnormalities. While some abnormalities aligned with the provided clinical history, most were incidental and identified in regions outside the primary area of interest.

## Discussion

### Management and policy variability of scout images

The findings of this study highlight a lack of uniformity in how scout images are managed across UK radiology departments. While many cross-sectional radiographers reported that their departments store scout images alongside the diagnostic series, others indicated that only the diagnostic images are stored, or that both the diagnostic series and scout images are stored, depending on the type of examination. Several respondents also noted the presence of informal procedures, yet were unsure whether formal departmental policies existed. This inconsistency reflects a broader gap in understanding and implementing professional guidance, reinforcing the need for clearer, standardised policies within and across departments.

These inconsistencies are also evident in how scout images are transferred and archived. Data storage plays a crucial role in medical imaging [26], and the RCR’s code of practice advises on consistency in image acquisition and storage [27]. Although most departments rely on PACS for storing and sharing imaging data [28], local practices regarding the duration of archiving scout images differ considerably, which could be due to PACS storage limitations and/or the higher cost of acquiring larger PACS storage space. The literature suggests that pressures on storage capacity continue to influence departmental decisions, with some centres omitting key imaging elements such as multiplanar reconstruction to reduce data volume [29, 30]. This aligns with participants’ uncertainty about the retention period for scout images, despite national guidance indicating that retention should be consistent across all records and imaging. The highlight of this uncertainty is limited awareness of scout image governance requirements and of how local policies align with national expectations.

Furthermore, variations in storage, transfer, and policy awareness may have direct implications for clinical decision-making. Inconsistent management practices may reduce the accessibility of scout images when they are needed to support the interpretation of subsequent findings [31]. There is therefore a clear need for better education, clearer local protocols and more transparent guidance to ensure that scout images are managed consistently across UK radiology departments.

### Awareness of the position statement from the CIB

This study also found considerable variation in awareness of the 2021 joint position statement on scout image management issued by the CIB. While some radiographers referred to existing departmental procedures, many were uncertain whether formal policies were in place, with 10 out of 19 radiology managers/leads expressing this uncertainty. The limited familiarity with national guidance observed in this study suggests that these concerns are not consistently recognised across departments. Limited awareness of national, regional and local policies highlights the need to explore how policies are disseminated and implemented across radiology departments.

Previous literature has highlighted the risks associated with inconsistent review of scout images, including potential medico-legal consequences [17, 19, 32]. Daffner [33] argued that radiologists could be held liable if a patient suffers harm due to neglecting to review scout images. Although any legal repercussions may arise from the clinical diagnosis and management, the cascading effect could also increase the likelihood that the radiographer will be held accountable for failing to send scout images to PACS for review. The CIB noted these instances in its joint position statement [20], emphasising the need for local policies on the transfer and review of scout images to safeguard both practitioners and patients. Participants’ reflections in this study support these concerns, with some radiographers demonstrating a willingness to learn more about the national policy. However, a well-defined local policy derived from the national consensus statement would provide radiographers with a deeper understanding of the importance of managing scout images and ensuring compliance with established protocols while clarifying the shared responsibilities of radiographers and radiologists.

### Diagnostic and clinical value of scout images

Scout images often reveal abnormalities that influence both diagnostic procedures and clinical decisions. The findings from this study indicate that incidental findings, such as malignant tumours, abdominal aortic aneurysms, and intra-abdominal haemorrhages, were often identified on CT and MRI scout images. This reflects the broader clinical value of scout imaging reported in the literature, particularly regarding asymptomatic incidental findings that may require urgent consultation with the reporting radiologist and a faster diagnostic pathway [34]. In some cases, abnormalities were described as being more apparent on scout images than on the diagnostic images due to technical factors such as saturation bands [35]. Such observations prompt adjustments to scan protocols or timely communication of concerns to the reporting radiologists, thereby supporting the efficiency of the diagnostic process.

This corroborates previous studies, such as that by Johnson and colleagues [36], where 2031 CT scout images were examined independently of the initial CT findings and patient history. They discovered that, of the total 18% abnormalities, 8.5% were visible on scout images but not on the actual axial images, and 2% were missed pathologic findings. While 2% may seem small, this suggests that 1.7 million of the 85 million CT scans performed annually in the United States could miss abnormalities visible only on scout images. Other studies [34, 35, 37, 38] reported similar findings, supporting the diagnostic value of scouts beyond traditional localisation. Collectively, these findings reinforce the clinical importance of reviewing scout images routinely and ensuring that radiographers understand their diagnostic potential beyond sequence planning and localisation.

### Implications for practice and recommendations

Scout images are vital components of cross-sectional imaging that require proper management, as they have the potential to reveal critical findings, whether suspected or incidental, that influence diagnostic pathways. However, the lack of clear, standardised policies/protocols for managing scouts, as well as radiographers’ lack of awareness of the importance of these images, compromises the quality of care and threatens patients’ quality of life.

Consideration should be given to educating cross-sectional imaging radiographers on the significance of scout images to enhance their understanding of the subject. This can be achieved through continuous professional development activities, including educational talks and discussions. Furthermore, all cross-sectional imaging departments in the UK and internationally should implement clear, agreed-upon protocols and policies for managing scout images. These protocols should specify actions to be taken in the event of unexpected findings or if an examination is aborted after scout image acquisition. Regular audits should be enforced to ensure departments adhere to these policies. Additionally, international research on the management of scout images could provide insights into protocols used in other countries.

### Strengths and limitations

We acknowledge the strengths and limitations of this study. To the best of our knowledge, this is the first survey to examine the existence of policies and protocols for managing scout data among a cohort of cross-sectional radiographers. A key limitation of the study relates to the sampling strategy. The study did not employ purposive sampling in its strictest sense, where participants are intentionally selected to capture narrowed experiences specifically focussed to the research question. Participants were recruited based on predefined but broad inclusion criteria; thus, the obtained sample may not have reflected the full range of relevant perspectives and characteristics within the target population. This may have reduced the depth of viewpoints represented, and therefore, the findings should be interpreted as exploratory. Additionally, the higher representation of respondents from England and the modest sample size of this study may limit generalisability. However, as Biau and colleagues [39] noted, a larger sample size that includes Radiologists could yield more significant findings. Of note, as with many online surveys [40, 41], the response rate could not be precisely determined, and non-response bias cannot be ruled out. Nonetheless, the findings provide an essential foundation for further enquiry and policy development. Future research could strengthen this area by using a more clearly defined purposive strategy.

## Conclusion

This study reinforces the importance of scout images as a vital component of cross-sectional imaging and highlights ongoing inconsistencies in their management across UK radiology departments. The absence of standardised policies and limited awareness of national guidance contribute to variable practice and potential gaps in patient safety. Although some radiographers demonstrate good understanding and adherence to local procedures, many rely on personal judgement due to the lack of formal policies/protocols. Strengthening policy frameworks and promoting education on the clinical value of scout images are essential to ensure consistent practice. A more unified approach would enhance diagnostic accuracy and improve the overall quality of patient care.

## Data Availability

All data generated or analysed during this study are available from the corresponding author upon request.

## Abbreviations

ACR: American College of Radiology
CIB: Clinical imaging board
CoR: College of Radiographers
CT: Computed tomography
IPEM: Institute of Physics and Engineering in Medicine
MRI: Magnetic resonance imaging
NHS: National Health Service
PACS: Picture archiving and communication systems
RCR: Royal College of Radiologists
SoR: Society of Radiographers
UK: United Kingdom

## Declarations

### Study design

Cross-sectional survey.
